# Single Cell Gene Expression Analysis in a 3D Microtissue Liver Model Reveals Cell Type-Specific Responses to Pro-Fibrotic TGF-β1 Stimulation

**DOI:** 10.3390/ijms22094372

**Published:** 2021-04-22

**Authors:** Catherine Jane Messner, Lmar Babrak, Gaia Titolo, Michaela Caj, Enkelejda Miho, Laura Suter-Dick

**Affiliations:** 1 School of Life Sciences, University of Applied Sciences and Arts Northwestern Switzerland, 4132 Muttenz, Switzerland; lmar.babrak@fhnw.ch (L.B.); gaia.titolo@gmail.com (G.T.); michaela.caj@fhnw.ch (M.C.); enkelejda.miho@fhnw.ch (E.M.); laura.suterdick@fhnw.ch (L.S.-D.); 2 Swiss Centre for Applied Human Toxicology (SCAHT), 4055 Basel, Switzerland; 3 SIB Swiss Institute of Bioinformatics, 1015 Lausanne, Switzerland; 4 aiNET GmbH, 4056 Basel, Switzerland

**Keywords:** single cell sequencing, in vitro, liver, liver fibrosis, liver microtissues

## Abstract

3D cell culture systems are widely used to study disease mechanisms and therapeutic interventions. Multicellular liver microtissues (MTs) comprising HepaRG, hTERT-HSC and THP-1 maintain multicellular interactions and physiological properties required to mimic liver fibrosis. However, the inherent complexity of multicellular 3D-systems often hinders the discrimination of cell type specific responses. Here, we aimed at applying single cell sequencing (scRNA-seq) to discern the molecular responses of cells involved in the development of fibrosis elicited by TGF-β1. To obtain single cell suspensions from the MTs, an enzymatic dissociation method was optimized. Isolated cells showed good viability, could be re-plated and cultured in 2D, and expressed specific markers determined by scRNA-seq, qRT-PCR, ELISA and immunostaining. The three cell populations were successfully clustered using supervised and unsupervised methods based on scRNA-seq data. TGF-β1 led to a fibrotic phenotype in the MTs, detected as decreased albumin and increased αSMA expression. Cell-type specific responses to the treatment were identified for each of the three cell types. They included HepaRG damage characterized by a decrease in cellular metabolism, prototypical inflammatory responses in THP-1s and extracellular matrix remodeling in hTERT-HSCs. Furthermore, we identified novel cell-specific putative fibrosis markers in hTERT-HSC (*COL15A1*), and THP-1 (*ALOX5AP* and *LAPTM5*).

## 1. Introduction

The generation of 3D-cell culture systems encompassing multicellular interactions has enabled the generation of in vitro liver models that retain in vivo like properties and many physiological functions. Existing 3D-liver models vary regarding cell types, media, 3D-architecture and flow conditions. These systems have conclusively demonstrated tremendous improvement in the ability to mimic and predict liver disease, hepatic metabolism, hepatotoxicity of drugs as compared to 2D, monocellular in vitro models [1]. Spheroids are a one of the most ubiquitous 3D-systems applied to the culture of liver cells and may be composed of primary or established cell lines. Either way, they are often utilized as multicellular systems of varying complexity, ranging from hepatocyte-only systems; to combination of hepatocytes with one or several types of hepatic non-parenchymal cells. Hepatocyte-only systems can be successfully be used to address hepatic metabolism. Whole genome analysis of primary human hepatocytes (PHHs) and two commonly used cell lines HepaRG and HepG2, has demonstrated that genes encoding drug-processing proteins are transcribed at a more similar level in HepaRG cells than in HepG2 when compared to both PHHs and liver samples [2,3]. Furthermore, HepaRG maintained in 3D-spheroids showed albumin production levels and CYP activity more similar to the liver when compared with 2D-monolayers [4]. This demonstrates the potential that HepaRG in a 3D system holds for producing human relevant data surrounding metabolism and hepatocellular injury.

For diseases and events that involve other actuators than the hepatocyte, multicellular systems are required. During the development of liver fibrosis, although the hepatocyte is the primary target of cellular injury, additional events are required including the activation of Kupffer cells and stellate cells [5]. 3D in vitro models of liver fibrosis have been developed with mouse or human primary cells, as well as with human cell lines. These systems are composed of several cell types in a self-assembled spheroid or microtissue, which develops the intercellular interactions required for the development of the disease phenotype [6,7,8]. In vitro, fibrosis can be elicited by exposing cells to the pro-fibrotic stimulus TGF-ß1. This cytokine is involved in the fibrotic adverse outcome pathway and is widely known to have strong pro-fibrotic effects in vivo and in vitro [5,9]. The effects of TGF-ß1 on the inflamed liver is widespread and multifactorial: it helps perpetuation of fibrosis by enhancing survival of myofibroblasts through inhibition of apoptosis and proliferation of activated hepatic stellate cells (HSC) [10] but also modulates sinusoidal endothelial cells and immune cells during the recovery from acute liver injury [11].

Previous publications have shown that a microtissue consisting of three cell lines HepaRG, THP-1, and hTERT-HSC can display a fibrotic phenotype when confronted with specific stimuli [8,12,13]. However, until now the response could only be assessed as a whole microtissue, without being able to discern between the contributions of each individual cell type. Due to its inherent complexity, changes in cell viability, cytokine production, or gene expression caused by the treatment cannot be assigned to a specific cell type.

Modern single cell sequencing (scRNA-seq) technologies provide us with a promising tool for determining cell specific gene expression and gene expression changes associated with a given disease phenotype. It has significantly improved the resolution of gene expression analysis in comparison to traditional sequencing, therefore providing greater scientific insights into complex multi-cellular systems [14]. This has been recently demonstrated by the successful evaluation of the heterogeneity of resting hepatic stellate cells and activated myofibroblasts [15]. Therefore, we hypothesized that a similar approach may provide insights on the individual responses of the cell types involved in the process of liver fibrosis (e.g., hepatocytes, Kupffer cells, and hepatic stellate cells). Several new platforms for scRNA-seq have been developed in the last few years providing scientists with an opportunity to further understand and visualize the heterogeneity in expression patterns between the same or different type of cells allowing us to measure cell to cell variability.

Efficient dissociation of complex systems such as tissues or multicellular spheroids can be technically challenging. Mechanical and/or enzymatic protocols can cause cellular damage and have been shown to alter gene expression if performed too vigorously or for too long [16]. Therefore, identifying an appropriate methodology for dissociation is critical. Regardless of the method employed, there are three criteria for adequate tissue dissociation: (1) high cell recovery; (2) preservation of cell integrity and functionality; (3) a simple and reproducible technique. For methods such as fluorescent assisted cell sorting (FACS) and scRNA-seq, it is imperative to dissociate microtissues into a single cell suspension without impairing cellular integrity and with minimal changes in gene expression. Commonly used tissue dissociation methods rely on enzymatic digestion, mechanic disruption, or a combination of both. Mechanical methods consist mainly of vigorous agitation on a vortex mixer [17], repeated pipetting, or specific dissociation devices [18]. Enzymatic dissociation, typically using collagenase mixtures, has been successfully applied for single cell sequencing [14,16]. Using liver tissue, MacParland et al. were able to obtain a single cell suspension and identify discrete cell populations. With this, they studied the hepatic immune microenvironment, demonstrating the potential of single cell resolution in understanding liver function and disease [14]. Although human-relevant data that can be obtained using human liver samples and scRNA-seq, this approach has several drawbacks. There are limitations in accessing human-derived material. Donor-to-donor variability also negatively influences the reproducibility of the model. Alternatively, suitable in vitro models with cell lines are more readily available, provide reproducible data (i.e., no donor-specific variations), and have the potential to be used in high-throughput screening [19].

In this work, we aimed at evaluating cell-type specific responses of a multicellular hepatic 3D-system in the context of TGF-β1-induced fibrosis. To this end, we established a robust and reproducible protocol for the dissociation of microtissues into viable cells that could be phenotypically characterized. Single cell sequencing using 10X Genomics analyzed using Cell Loupe browser following a previously published approach [20,21,22] led to the identification of gene expression profiles characteristic of the three cell types included. Moreover, we were able to identify cell-type specific responses to the treatment, which unveiled molecular responses that could further improve current understanding of liver fibrosis.

## 2. Results and Discussion

The power of multicellular, 3D-culture in vitro systems is that they can mimic complex biological process that require intercellular interactions. MTs faithfully recapitulate human physiology and are commonly used as an in vitro tool in a range of scientific subjects such as developmental, disease, and toxicological research [23]. More specifically, 3D human liver models provide more relevant data than traditional 2D culture methods [1]. However, the identification of individual cell type responses in compact, tissue-like 3D structure is challenging, as single cell suspensions are necessary for techniques such as FACS or scRNA-seq.

Here, we set out to isolate cells from 3D-liver MTs consisting of three cell types and to identify their individual responses to the pro-fibrotic cytokine TGF-β1. We successfully established a reproducible protocol for the dissociation of multicellular hepatic MTs that did not cause cellular (oxidative) stress or affect the maintenance of important cellular characteristics, such as attachment, production of albumin or responsiveness to TGF-β1.

### 2.1. Microtissue Dissociation

**Figure 1:**
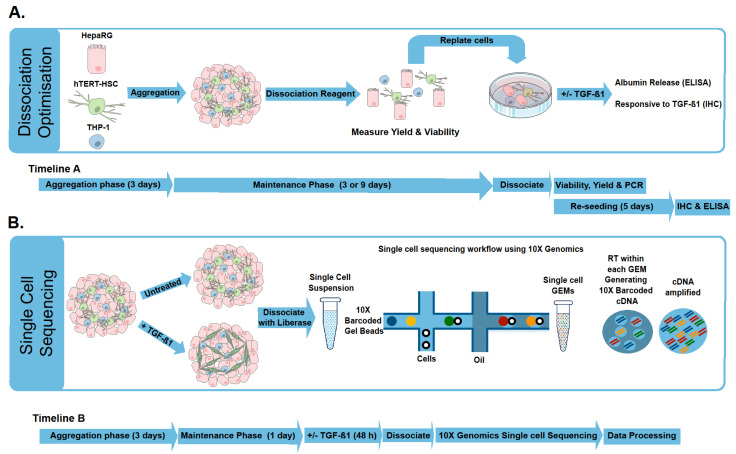
Graphical representation of experimental methodology with corresponding timelines. Human liver microtissues (MTs) are generated using HepaRG, hTERT-HSC, and THP-1. Initial experiments focused on optimization of dissociation using Liberase, Accutase and Accumax by comparing yield and viability. After dissociation, cells were suspended in 96 well plates to assess the maintenance of crucial cellular characteristics: HepaRG were able to release albumin; hTERT-HSC were able to become activated upon exposure to TGF-β1 (**A**). The optimized dissociation protocol was used to dissociate TGF-β1 treated and untreated MTs for processing following the single cell sequencing workflow (**B**).

**Figure 2:**
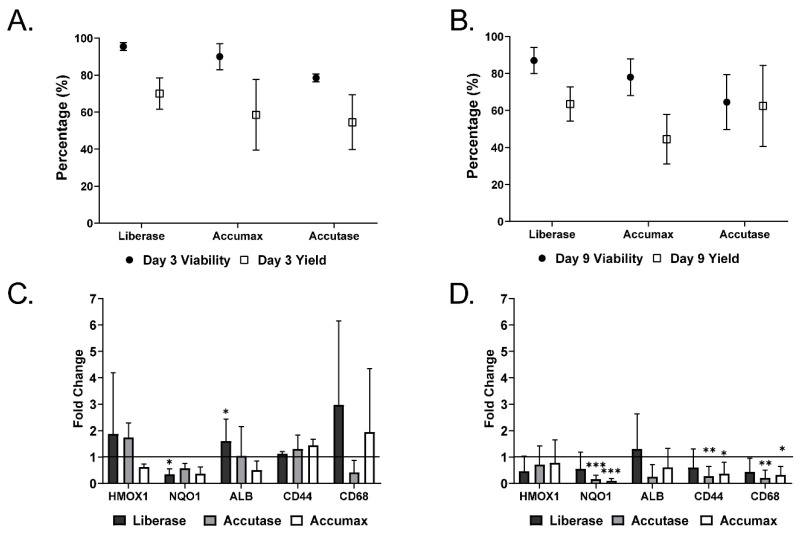
Cell dissociation outcome. MTs were generated using differentiated HepaRG, differentiated THP-1 and hTERT-HSCs. MTs were allowed to aggregate over 72 h and then maintained in FBS-free medium for 3 or 9 days. Dissociation was carried out using Accutase, Liberase or Accumax at day 3 (**A**) and 9 (**B**) of maintenance. Trypan blue was used to assess the percentage of viability of the cells immediately after dissociation and cell number was recorded in order to calculate the cell yield percentage from the MTs by comparing cell number following dissociation to cell number used to generate the MTs. Stress markers (HMOX1 and NQO1) and hepatic markers (ALB, CD44 and CD68) were investigated using q-RT-PCR directly after dissociation at day 3 (**C**) and day 9 (**D**) and compared to undissociated MTs (baseline). Data represent mean ± SD (*n* = 6). Statistical analysis using unpaired student’s *t*-test; *: *p* ≤ 0.05; **: *p* ≤ 0.01; ***: *p* ≤ 0.001.

Dissociation of MTs without affecting gene expression or eliciting cellular stress can be problematic, therefore, an efficient and reproducible method resulting in high yield, viability and maintenance of cellular characteristics is required [16]. Mechanical dissociation is a rapid technique that works well on certain samples. However, yield and viability can be inconsistent and low [24]. It has been suggested by many publications that MTs show increased physiological relevance due to cell-cell adhesion and interactions [25,26] and the production of ECM [27], suggesting that mechanical dissociation may not be adequate or enough for MT dissociation. As depicted in Figure 1, MTs consisting of HepaRG, hTERT-HSC and THP-1 were generated, maintained and ultimately dissociated 3 or 9 days after aggregation. The three enzymatic dissociation protocols tested (Accutase, Accumax and Liberase) were able to dissociate the MTs into single cell suspensions, but the incubation with 1.3 U/mL Liberase at 37 °C, 5% CO_2_ for 40–60 min gave the best results. Approximately 60–70% of the cells in the MTs could be recovered (yield) and their viability was greater than 85% (Figure 2A,B). Enzymatic digestion also did not cause oxidative stress, as expression levels of HMOX1 and NQO1 were decreased by the process (Figure 2C,D). Liberase significantly decreased NQO1 for day 3 dissociated MTs, whereas Accutase and Accumax significantly decreased NQO1 expression for day 9 dissociated MTs. However, dissociation with Accutase and Accumax also led to a reduction in expression of cell-specific markers albumin (ALB), hyaluronic acid receptor (CD44) and CD68 in MT dissociated on day 9 (Figure 2D). Dissociation with Liberase, on the other hand, did not significantly affect the expression of these markers. It has been demonstrated by Waise et al. and MacParland et al. that by using collagenase as the enzyme of choice on tissues, it is possible to obtain a single cell suspension suitable for scRNA-seq [14,16]. Waise et al. also assessed the suitability of other enzyme mixtures and our findings are in agreement that 15 min incubation with Liberase TL (due to its gentle properties) is not sufficient to allow full dissociation [16]. However, enzymatic dissociation use Liberase TL has been used previously on spheroids to obtain single cell suspensions that could be successfully re-seeded following the disaggregation process [28]. Thereby, increasing the incubation time to 40 min or 60 min at day 3 and 9, respectively, was sufficient to obtain a single cell suspension, while maintaining high viability and yield and without eliciting increases in stress or loss of cellular characteristics. This optimized method was also suitable for dissociating TGF-β1 treated MTs, thereby allowing the comparison of treated and untreated MTs using scRNA-seq as described below.

**Figure 3:**
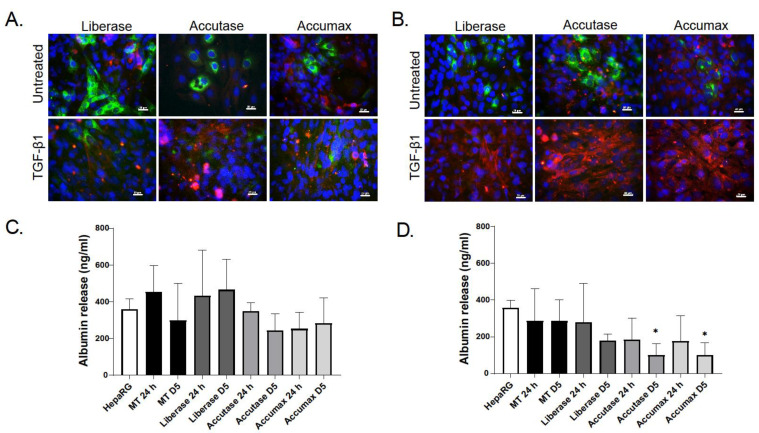
Cellular characteristics and functionality of cells obtained by dissociation of MTs. MTs were dissociated at day 3 (**A**) or 9 (**B**) using Accutase, Liberase or Accumax. Isolated cells were seeded directly into a 96-well and allowed to attach for 3 days. Replated cells were treated with TGF-β1 (1 ng/mL) for 48 h. Activation of hTERT-HSCs was determined by α-SMA immunostaining and functionality of HepaRGs by albumin immunostaining. Staining for albumin is in green, α-SMA in red and DAPI in blue. Photomicrographs are taken at 40× magnification scale bar is 20 µm. Albumin secretion of cells dissociated at day 3 (**C**) and day 9 (**D**) was determined 24 h and 5 days after re-plating using an ELISA. Data are expressed as albumin concentration ± SD of *n* = 6–9. Statistical analysis using unpaired student’s *t*-test comparing dissociated cells to MT for each time point; *: *p* ≤ 0.05.

Cells dissociated with Liberase were not only viable, but also able to attach to cell plates and display basic functional characteristics. Recovered and re-plated cells expressed and secreted albumin, as detected by immunostaining and ELISA (Figure 3). They also retained the ability to respond to TGF-β1 (1 ng/mL) stimulation, as shown by the decrease in albumin expression and the increase in the expression of α-SMA, that correspond to hepatocellular injury and HSC activation, respectively. Both are known events that occur during liver fibrosis progression [29,30,31]. In contrast, dissociation of MTs with Accutase and Accumax led to a lower number of functional HepaRGs, as indicated by the lower number of albumin positive cells following day 3 dissociation (Figure 3A). Similarly, Accumax dissociated cells at day 9 also resulted in lower numbers of albumin-positive cells (Figure 3B). The dissociation process did not result in spontaneous activation of hTERT-HSCs as demonstrated by low αSMA levels. Subsequent experiments were performed using Liberase for the enzymatic digestion.

### 2.2. Characterization of Dissociated Cells

**Figure 4:**
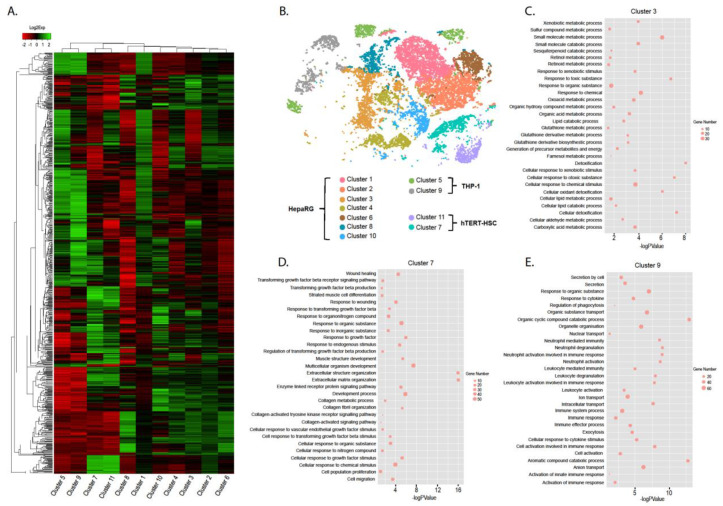
Graph-based clustering of untreated MTs to identify cell sub-groups. Single cell sequencing data was evaluated using Graph-based unsupervised clustering. Cells could be grouped in 11 clusters represented in a heatmap depicting differentially expressed genes for each cluster (**A**). Clusters 1 to11 are shown in a tSNE plot with the clusters grouped into the corresponding cell type (**B**). The identified differentially expressed genes for each cluster were analyzed regarding biological processes using Gene Ontology (GO; http://geneontology.org/, accessed on 20 January 2021). Examples of the biological pathways from three clusters are depicted: Cluster 3 is an example of HepaRG (**C**); Cluster 7 is an example of hTERT-HSC (**D**); Cluster 9 is an example of THP-1 (**E**).

Using graph-based unsupervised clustering, 11 clusters were identified and successfully grouped into the three cell-types used to generate the MTs. The results depicted as a heatmap demonstrate that three separate groups can be distinguished based on the gene expression patterns: group 1 consisting of cluster 5 and 9; group 2 consisting of cluster 7 and 11; group 3 consisting of clusters 1–4, 6, 8 and 10 (Figure 4A,B). The identity of each group was assigned based on the transcriptional profiles of the clusters and the corresponding GO terms identified (Supplementary data) in accordance with known liver functions [32]. Group 1 were identified as THP-1 cells due to the overwhelming number of inflammatory and secretory biological pathways associated with this group (Figure 4E). Group 2 was confirmed to be hTERT-HSC based on the large number of biological pathways associated with ECM remodeling, wound healing and TGF-β signaling (Figure 4D). Finally, HepaRG were identified based on their GO terms associated with glutathione activity, detoxification and response to xenobiotics (Figure 4C).

Using putative markers of HepaRG, hTERT-HSC and THP-1, we were also able to successfully identify three cell-specific clusters within the scRNA-seq data that correlated strongly with the graph-based clustering which will be further described in the subsequent section. From the graph-based clustering and deeper analysis of the scRNA-seq data, we see potential similarities between some of the HepaRG clusters and the different zones within the human liver. The clusters, within each cell-type, were compared to identify significantly higher expressed genes in comparison to the other clusters within that group. The clusters characteristic of THP-1 (cluster 5 and cluster 9) and the two assigned hTERT-HSC (cluster 7 and cluster 11) showed no notable differences with regards to gene ontology (Supplementary data). Within the liver, metabolic zonation occurs due to a variety of conditions and signals including oxygen, nutrient, metabolites, hormones and cytokine gradients resulting in hepatocytes that are functionally different dependent on location [33]. Concordantly, we also identified sub-groups within the HepaRG cluster in the MTs. The significant increase in specific genes identified in Cluster 1 and 10, suggest the function of these cells are similar to the hepatocytes found in the pericentral zone in the liver. Cluster 1 expresses lactoylglutathione lyase (*GLO*), which is involved in the detoxification of a toxic bi-product of glycolysis, methylglyoxal (MG) [34], suggesting that the glycolysis pathway could be active in these cells. Furthermore, recent a publication also using scRNA-seq has demonstrated that pericentral hepatocytes express a large number of ribosomal related mRNAs [35], which was also found for cluster 1. In cluster 10, we find increased *HILPDA* expression, which is involved in regulating lipid droplet formation and triglyceride storage in hypoxic conditions [36,37]. Furthermore, stabilized and active β-catenin is also a pericentral hepatocyte characteristic and *CA9*, which is significantly higher in cluster 10, is involved in the stabilization of cytoplasmic β-catenin [38]. Contrarily, cluster 3 expresses markers associated with periportal hepatocytes, including expression of keratin 19 (*KRT19*) [39]. Furthermore, *NNMT* expression was also significantly increased in cluster 3, which suggests that there is increased gluconeogenesis (another periportal characteristic). Previous evidence suggests that *NNMT* regulates gluconeogenesis in hepatocytes [40]. Interestingly, ferritin light chain (*FTL*) was also significantly increased in cluster 3 and liver iron concentrations have been shown to be higher in the periportal region [41]. Clusters 2, 4, 6 and 8 had a mixed gene expression phenotype.

**Figure 5:**
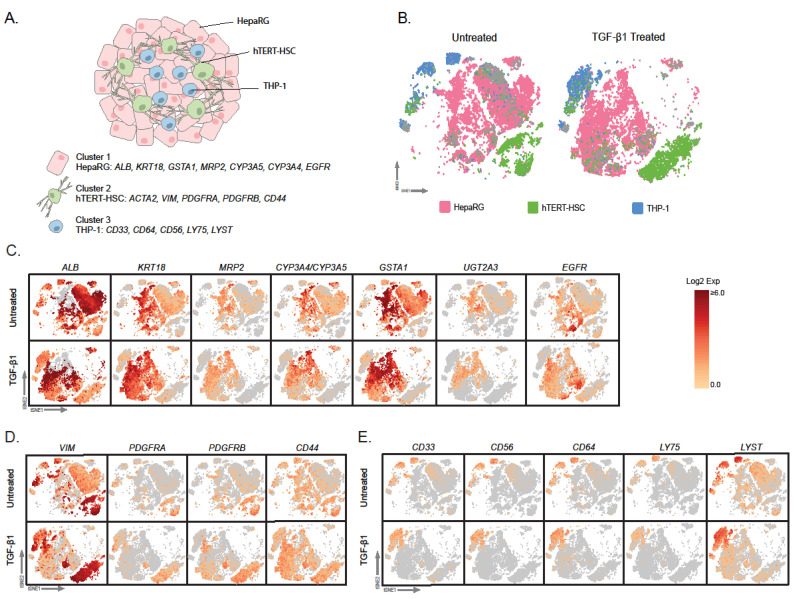
Expression of cell-specific markers in the identified cell clusters. Pre-processed single cell sequencing data was analysed using CellLoupe. Schematic representation of the composition of multicellular MTs and known cell type-specific markers (**A**). Supervised clustering based on the cell type-specific markers in untreated and TGF-β1-treated samples led to the identification of HepaRG (pink), hTERT-HSC (green) and THP-1 (blue) and cells that did not cluster based on the chosen genes (grey) more detail can be found in methods section “Gene-based identification of specific cell clusters“ (**B**). Examples of marker expression for HepaRG (**C**), hTERT-HSC (**D**) and THP-1 (**E**) in untreated and TGF-β1-treated samples. As *ACTA2* is also a fibrotic marker it is shown in Figure 7. Data are expressed as log2 expression (selected markers shown in tSNE plots).

In order to streamline the analysis of TGF-β1 treated and untreated MTs, three clear clusters were identified using a panel of putative markers (gene list and literature in Figure 5A) for HepaRG (Figure 5C), hTERT-HSC (Figure 5D) and THP-1 (Figure 5E). They were used to identify expression patterns and cellular localization within the tSNE plot from the single cell sequencing data (Figure 4). Furthermore, TGF-β1 treated MT data was included to demonstrate the location of the cell types in both conditions. Two hepatocytic markers *ALB* and *KRT18* were preferentially expressed in the central cluster. This area was also positive for the canalicular organic anion exporter *MRP2*, phase I metabolic enzyme *CYP3A4/3A5* and phase II enzymes *GSTA1* and *UGT2A3* (Figure 5C). Stellate cell markers *VIM, PDGFRA* and *PDGFRB* were positively expressed mostly in the lower right cluster (Figure 5D). THP-1 have been shown to express *CD33, CD56* and *CD64* and these markers localized to the upper left cluster (Figure 5E). A small group of cells were left remaining (in grey) which did not associate with any of the markers and were not included in the differential gene expression analyses. However, due to their proximity to the HepaRG cluster, it is likely they are a sub-group of HepaRG (Figure 5B).

In summary, using scRNA-seq, we are able to discern the three cell types included in the MTs. Moreover, within the HepaRG cluster, we identified subgroups expressing specific markers that have been associated either with periportal or pericentral hepatocytes. This may indicate phenotypic differences of the HepaRG within the MT and suggests that MTs may reflect the physiology of the liver, as cells in 3D are also subjected to gradients in oxygen and nutrients.

### 2.3. Effect of TGF-β Treatment on Multicellular MTs

**Figure 6:**
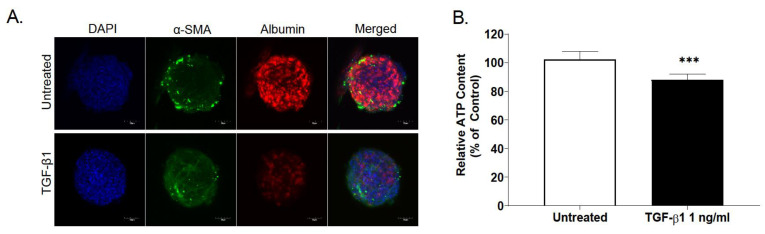
Effects of the pro-fibrotic cytokine TGF-β1 on human liver microtissues. MTs were exposed to TGF- β1 (1 ng/mL) for 48 h. Immunostaining of treated and untreated MTs was carried out: DAPI (blue), α-SMA (green) and albumin (red), 20× magnification (**A**), scale bar is 50 µm. Albumin staining was used to assess HepaRG function, whereas the formation of α-SMA fibers indicate the activation of hTERT-HSC in the treated MTs. CellTiter-Glo^®^ Luminescent Cell Viability Assay was used to measure relative ATP of TGF- β1 treated MTs in comparison to untreated. Data represent mean ± SD (*n* = 9) (**B**). Statistical analysis using unpaired student’s *t*-test: ***: *p* ≤ 0.001.

Treatment of MTs with the profibrogenic TGF-β1 (1 ng/mL) for 48 h resulted in activation of hTERT-HSC evidenced by increased αSMA stress fibers and decreased albumin production in comparison to the untreated MTs (Figure 6A). In addition, TGF-β1 treatment also led to a significant decrease in viability (−11%) and a reduction in albumin immunostaining (Figure 6). The response to TGF-β1 treatment did not hinder the dissociation of the MTs using the optimized procedure with Liberase. We could recover 66% from the cells from the treated MTs, displaying a viability of 86%. These values are only slightly lower than the ones obtained with untreated MTs, with a yield of 71% and a viability of 95%.

**Figure 7:**
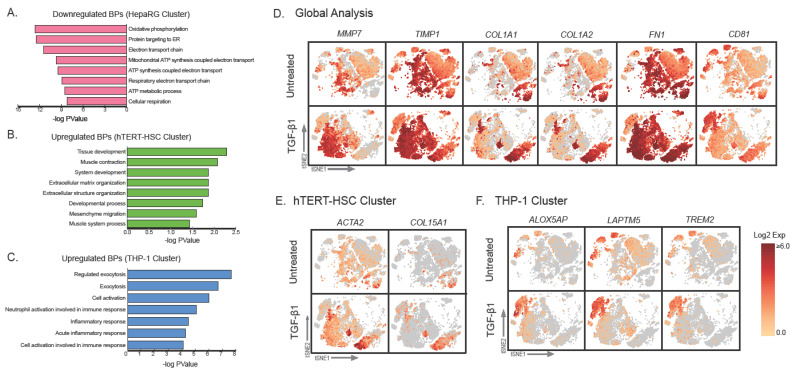
Visualization of fibrosis-relevant markers Specific effects on genes of interest are shown based on the scRNA-seq data to identify cell-specific responses. The responses of individual cell clusters (supervised method) to TGF-β1 (1 ng/mL) treatment for 48 h were evaluated to identify treatment-related cell specific changes in gene expression. Significantly differentially expressed genes (LogFC *p* < 0.05) for each cell type were identified and using gProfiler to identify the corresponding biological pathways and expressed as −log (*p* Value). A total of 48 significantly downregulated genes were identified for treated HepaRG (**A**), 25 significantly upregulated genes for hTERT-HSC (**B**) and 45 significantly upregulated for THP-1 (**C**). Graphs D–F represent expression changes of specific genes: Genes that were significantly regulated in the global TGF-β1 treated sample vs. untreated (**D**) treated hTERT-HSC cluster vs. untreated hTERT-HSC cluster (**E**) and THP-1 cluster vs. untreated THP-1 cluster (**F**). Data are expressed as log2 expression (selected markers shown in tSNE plots).

To assess the effect of TGF-β1 on the individual cell types present in the MT, the single cell suspension from treated and untreated samples were processed using 10X genomics technology and sequenced. Data confirmed expected responses from TGF-β1 exposure such as significant increases in *FN1, TIMP1, MMP7, COL1A1* and *COL1A2* expression and a significant decrease in *ALB* expression (Figure 5C and Figure S1). These changes in gene expression are in accordance with key events involved in liver fibrosis [5]. Furthermore, cell-specific changes (e.g., increased inflammation in TGF-β1 treated THP-1) are also shown using gProfiler results (Figure 7A–C). tSNE plots depict the specific location of the genes that were upregulated in the treated samples in comparison to the untreated (Figure 7D–F) in the context of the cell-type specific clusters defined in Figure 5B. The ECM components *COL1A1* and *COL1A2* were expressed in lower amounts throughout the HepaRG and THP-1 clusters, and as expected the highest expression was located in the hTERT-HSC cluster. Interestingly, *FN1* was strongly expressed by both HepaRG and hTERT-HSCs (Figure 7D).

We were able to identify more specific changes occurring within the cell clusters upon exposure to TGF-β1 (Supplementary data). TGF-β1 treated HepaRG were compared to untreated HepaRG and 48 significantly downregulated genes were identified and associated to oxidative phosphorylation, electron transport chain and ATP synthesis (Figure 7A). Decreased ATP production could be linked to the HepaRG being damaged as a consequence of TGF-β1 treatment and reduced electron transport chain activity has previously been linked to other liver conditions such as biliary cirrhosis [42]. HepaRG could also have undergone a respiratory shift to glycolysis as oxidative phosphorylation is decreased, which can also occur during liver fibrosis [43].

TGF-β1 treatment elicited a significant increase for 25 genes in hTERT-HSC. The highest increase was seen for *ACTA2* (αSMA), which was not detected through global analysis (Figure 7E). This is likely due to scRNA-seq taking the average cell count of the gene across the whole sample, obscuring hTERT-HSC gene expression due to the higher number of HepaRG cells in each MT. As expected, gProfiler results demonstrated an increase in biological processes (BPs) corresponding to tissue development, extracellular matrix (ECM) organization and ECM structure organization (Figure 7B). In addition, using the scRNA-seq, we were able to identify a significant increase in *COL15A1* (Figure 7E), which was not identified using the global comparative methods. There are links between increased *COL15A1* and liver fibrosis progression [44]. *COL15A1* is a large fibrillar collagen and provide structural integrity to ECM [45], yet the link between *COL15A1* expression and HSC activation is poorly understood and warrants further investigation.

Finally, THP-1 cells treated with TGF-β1 resulted in 45 significantly upregulated genes. The BPs identified for these genes were associated with exocytosis, cell activation and inflammatory response (Figure 7C). This is in accordance with previous findings, as the Kupffer cells (KCs), which THP-1 cells represent in the MTs, are responsible for eliciting a pro-inflammatory response during fibrosis progression [5]. By focusing on the genes upregulated within the TGF- β1 treated THP-1 against the untreated THP-1 cluster, rather than global comparison, we were able to identify *TREM2*, *ALOX5AP* and *LAPTM5* that were significantly upregulated (Figure 7F). scRNA-seq of cirrhotic human liver samples has recently detected a subpopulation of *TREM2+CD9+* macrophages that expands during liver fibrosis [46]. In our model, we also see an increased *TREM2* expression in the TGF-β1 treated MTs specifically on the macrophages (Figure 7F), which indicates similarities between our 3D-MT model and in vivo findings. *ALOX5AP* has been shown to be expressed in KCs and is essential for their survival [47], is upregulated during early stages of fibrosis progression [48] and plays a role in promoting inflammation [49]. *LAPTM5* is also a positive regulator of pro-inflammatory signaling in macrophages and has been linked to non-alcoholic fatty liver disease in mice [50]. These could be interesting genes for further investigation and to elucidate their role in the liver KCs during fibrosis progression.

These findings are in agreement with the previous literature that highlight the benefits that can be obtained using the increased resolution that scRNA-seq provides when investigating multicellular systems [14]. The combination of the 3D-MT model and the analytical methodology represent a very valuable tool to investigate molecular processes involved in the development of liver fibrosis. However, some limitations of the system need to be taken into consideration, in particular the relevance to the disease development in the patient. The data reported here were obtained from an in vitro model that mimics many key aspects of liver fibrosis. It remains to be seen to what extent it reflects the patient, as a direct comparison with diseased liver tissue analyzed using a similar technological approach is lacking. This is mainly due to limited available data and the heterogeneity in methodology leading to technological noise. Subsequent studies including clinical samples analyzed in a comparable manner should address this question.

## 3. Materials and Methods

### 3.1. Generation of MT

Microtissues (MTs) were generated as described in Messner et al. 2019 [12] by combining three different cell lines representative of hepatocytes, Kupffer cells and hepatic stellate cells in the Sigma micro-mold system (Z764051-6EA; Sigma, St. Louis, MO, USA). Briefly, MTs were generated by self-assembly of differentiated HepaRG (Biopredic, Rennes, France), differentiated THP-1 (Cell Line Service) and hTERT-HSC (provided by Dr. Bernd Schnabl UC San Diego, CA, USA) at a ratio of 2:1:1. These cell-lines are surrogates for hepatocytes, Kupffer cells, and stellate cells, respectively. Aggregation medium was composed of William’s E Medium + GlutaMAX (Cat. No. 32551; Invitrogen), 2 mM l-glutamine (Cat. No. G7513; Sigma), 1X ITS (Cat. No. 11074547001; Sigma), 100 nM Dexamethasone (Cat. No. D1756; Sigma,), 20% fetal bovine serum (FBS), and 1% penicillin and streptomycin (P/S). Following 72 h of aggregation, medium was then exchanged to an FBS free version and the MTs were maintained for 3 or 9 days prior to dissociation.

### 3.2. Viability Assay

The cell viability for cell suspension after dissociation was assessed using Trypan blue exclusion dye (Sigma; T8154). The viability of MTs treated with TGF-β1 1 ng/mL and untreated was assessed using the CellTiter-Glo^®^ Luminescent Cell Viability Assay (Promega, Madison, WI, USA; G7570), and the luminescence was read at FlexStation3 for 1000 ms.

### 3.3. Immunostaining

Immunostaining was carried out for the cells seeded in a 96-well plate, following standard protocols. Cells were fixed with buffered 4% PFA for 20 min and permeabilized with 0.1% Triton X-100 for 15 min. Blocking was performed using 1% BSA for 1 h at room temperature (RT), and then cells were incubated with the primary antibody for 2 h at RT or overnight at 4 °C. Then the secondary antibody was applied for 1 h at RT (Table 1) and counterstained with DAPI for 5 min. In between every step, the cells were washed with 1x PBS containing Mg^+2^ and Ca^+2^ three times. Fluorescence images were taken using Axio Software SE64 Rel. 4.9.

### 3.4. Quantitative Real Time Polymerase Chain Reaction

RNA was extracted from liver MTs and the cell suspensions obtained following the MT dissociation protocol at day 3 following standard TRIzol procedure with the addition of glycogen (LT-02241; ThermoFisher, Waltham, MA, USA). Extracted RNA was reverse transcribed using a M-MLV Reverse transcriptase (M1705; Promega,), M-MLV RT buffer (M531A; Promega), dNTP Mix (02-31-00100; Solis BioDyne), and Oligo dT-Primer (79237; Qiagen). The quantitative real-time polymerase chain reaction (qRT-PCR) was carried out using TaqMan probes (Table 2) for selected oxidative stress markers (HMOX1 and NQO1) and hepatic markers characteristic of HepaRG surrogates for hepatocytes (ALB), hTERT-HSC (CD44) and THP-1 surrogates for KCs (CD68). FastStart TaqMan Polymerase (04673433001; Roche) was used to perform the qRT-PCR. Program settings: 10 min denaturation at 95 °C, followed by 40 cycles of 15 s at 95 °C and 1 min at 60 °C. The Ct values were generated using the Corbett Rotorgene Analysis Software 6000 and processed on GraphPad Prism. Gene expression changes were calculated using the ΔCt method with GAPDH as a house keeping gene. Fold changes were calculated as 2-(Δ(ΔCt) and expressed as mean and SD of 2 biological repeats with 3 replicates each.

### 3.5. Albumin Enzyme-Linked Immunosorbent Assay

Secreted albumin was determined in cell culture supernatant 1 and 5 days after MTs dissociation and cell re-plating in a 96 well plate. Albumin quantification was performed using the Human Albumin ELISA Quantitation Set (Bethyl Laboratories, Montgomery, TX, USA; E80-129) performed in high binding flat-bottomed plates (Greiner-bio one, Kremsmünster, Austria; 655 061), following provider’s instructions. Supernatant from HepaRG (20,000 cells/well) was collected after 5 days and used as positive control, while hTERT-HSC media was used as negative control. Absorbance at 450 nm was detected with the FlexStation3, Molecular Devices (Bucher Biotec AG, Basel, Switzerland) and albumin concentration was calculated based on a standard curve and applying a 4 parameter-fit on the SoftMax Pro software: Albumin secretion is expressed as quantity released per 24 h.

### 3.6. Dissociation of the Microtissues

To establish optimized dissociation conditions different approaches were carried out as summarized in Table 3. The MTs were collected in 900 μL PBS, washed and then resuspended in Liberase TL Research Grade™ 1.3 U/mL (Roche; 5401020001), Accutase™ (Invitrogen, Carlsbad, CA, USA; 00-4555-56) or Accumax (Invitrogen; 00-4666-569) using 1.5 mL Protein LoBind Tubes (Eppendorf; 022431081). The dissociation for the HepaRG, hTERT-HSC, and THP-1 MTs was carried out in an incubator for 40 to 60 min at 37 °C and 5% CO_2_ using the built-in shaker at 100 rpm. Following 20 min of incubation, the tubes were shaken by hand 10 times and the cycle of incubation and shaking was repeated until MT appeared visibly dissociated. Subsequently, the cell suspension was pipetted up and down an additional 10 times with a p1000 pipette. Dissociation status was assessed via microscopic observation. 600 μL of aggregation medium was added and the cell suspension was centrifuged at 400 RCF at 37 °C for 5 min and resuspended in 300 μL aggregation medium. The number of resuspended cells was determined in 30 μL and the remaining 270 μL were equally split in three aliquots and seeded in a 96 well plate for cell characterization. Cell recovery and morphology were observed using phase contrast microscopy 24 h, 3 and 5 days after dissociation. On day 3 after dissociation, replated cells were treated with TGF-β1 (Sigma; T5050, 1 ng/mL), for a further 2 days. Cell culture supernatant was collected at 24 h and day 5 for albumin ELISA and cells were fixed with 4% PFA on day 5 for subsequent immunostaining.

### 3.7. scRNA-Seq

Four untreated and four TGF-β1 treated samples were generated using 100 MTs per condition (≈200,000 cells per condition) and processed for 10× Genomics Chromium Next GEM Single Cell following the user guide. After dissociation of the MTs, cell number and viability were assessed, resulting in a 71% cell yield and 95% viability for the untreated samples and a 66% yield with 86% viability for the TGF-β1 treated samples. Of these dissociated cells, 17,400 individual cells per sample with a viability of 70% or higher were partitioned into the 10× Next GEM Chip and gel beads-in-emulsion (GEMs) were generated for a target capture of 10,000 cells per sample as described by the user guide. One untreated sample became clogged in the microfluidic device during processing and could not be moved forward for library preparation. After GEM generation, reverse transcription was performed using a Biorad PCR machine. cDNA was then recovered using 10X Genomics Recovery Agent and then cleaned by Silane DynaBeads. cDNA was then amplified for 11 cycles and then cleaned using SPRIselect beads (Beckman). cDNA libraries were prepared for Illumina sequencing as described in the user guide. Quality control and quantification steps indicated in the user guide were performed using the Bioanalyzer (Agilent Technologies). The remaining cell suspensions were collected and lysed using TRIzol for qRT-PCR based gene expression analysis.

### 3.8. Sequencing

The prepared libraries (total of 7) were sent to the Genomics Facility Basel at ETH and sequenced using the NovaSeq PE 28/91, for a total of 1.2–1.6 M reads. A sequencing depth of 200,000 sequences per cell was targeted. Raw sequencing data demultiplexed by the facility using Cell Ranger pipeline (Cellranger mkfastq v3.0) and aligned to a reference germline database (GRCh38-2020-A) using Cellranger count v5.0.

### 3.9. Data Analysis Using Cell Ranger Loupe Browser

To analyze all the samples together, the sample files were pooled together using the 10X Genomic’s Cellranger aggr pipeline to be analyzed via Cell Loupe Browser 5.0. The Cellranger aggr pipeline automatically equalizes the average read depth per cell between groups before merging. This approach avoids artifacts that may be introduced due to differences in sequencing depth. The gene expression from these data were filtered, normalized and clustered using Cell Loupe Browser. Briefly, in order to reduce the gene expression matrix, Cell Ranger performs Principal Component Analysis (PCA) to reduce the dimensionality of the dataset through num_principal_comps that uses a Python implementation of the IRLBA algorithm [51] and visualized using t-distributed Stochastic Neighbor Embedding of principal components (t-SNE). To robustly and confidently cluster the cells, we decided to cluster the cells using specific and verified gene markers for each cell-type (Section Gene-based identification of specific cell clusters).

Once the clusters were determined, Cell Ranger uses the sSeq method to find differentially expressed genes between clusters [52]. When the counts become large, Cell Ranger changes to an asymptomatic beta test used in edgeR [53]. For each cluster, the algorithm is run on that cluster versus all other cells, yielding a list a genes that are differentially expressed in that cluster compared to the rest of the sample. Instead of the sSeq’s implementation, Cell Ranger computes relative library size as the total UMI counts for each cell divided by the median UMI counts per cell. Similar to sSeq, normalization is performed by a per-cell library-size parameter that is incorporated as a factor in the exact-test probability calculations. To filter out multiplets, low quality cells, and empty droplets, filtering was performed as follows: UMIs were normalized to include counts between approximately 200 and 40,000, features included were in the range of 25–6000, the mitochondrial fraction percentage of UMIs per barcode associated with mitochondrial genes was set at 50%. The mitochondrial percentage is set higher than standard due to the higher mitochondrial gene content of hepatocytes [14] and 10 principal components were applied (default). This removed 887 (3% of barcodes (0.1–4.1% of each cell type)).

### 3.10. Gene-Based Identification of Specific Cell Clusters

Cells were clustered based on known markers for each cell type. Due to high specificity of THP-1 markers, they were clustered first using genes shown in Table 4 and grouped. HepaRG were grouped by the genes in Table 4 and the additional rule “not in THP-1 cluster”. Finally, hTERT-HSCs were clustered based on genes in Table 4 and the additional rule “not in THP-1 and not in HepaRG clusters”. Cells that did not express any of the genes listed below were classified as “other” and were not used in further analysis. To further investigate sub-groups within each cluster, we used graph-based unsupervised clustering.

## 4. Conclusions

In conclusion, TGF-β1 treated and untreated liver MTs could be dissociated and analyzed using scRNA-seq, thereby providing a higher resolution of cellular expression in comparison to classic sequencing. Effects of TGF-β1 on the MT as a whole confirmed that the MTs responded to the treatment and displayed decreased hepatocyte functionality (reduction in albumin expression), increased HSC activation (upregulation of α-SMA) and inflammatory responses. scRNA-seq allowed a granular analysis on a single cell level, demonstrating for the first time the maintained characteristics of each of the three cell types coexisting in a liver MTs as well as the cell-type specific responses to the pro-fibrotic TGF-β1. Moreover, the results indicate the existence of HepaRG subpopulations in the MT bearing similarities to hepatocytes from different zonal areas of the liver. Furthermore, we demonstrated that single cell sequencing helps identifying novel, cell-specific markers such as *COL15A1*, *ALOX5AP,* and *LAPTM5* that could further improve our understanding of liver fibrosis.

## Figures and Tables

**Table 1:** Antibodies used for immunostaining.

Protein of Interest	Primary Antibody	Secondary Antibody
Albumin	Monoclonal rabbit anti-albumin (EPR20195) 1:700 dilution (abcam, ab207327)	F(ab’)2-Goat anti-rabbit IgG (H + L) cross-adsorbed secondary antibody, AF488 conjugated, diluted 1:1000 (Invitrogen, A11070)
α-Smooth muscle actin (α-SMA)	Monoclonal mouse anti-α-SMA, 1:300 (Sigma, A5228)	F(ab’)2-Goat anti-mouse IgG (H + L) cross-adsorbed secondary antibody, AF546 conjugated, diluted 1:1000 (Invitrogen, A11018)

**Table 2:** List of TaqMan probes.

Marker of Interest	Abbreviation	Catalogue Number
Glyceraldehyde 3-phosphate dehydrogenase	GAPDH	Hs02758991_g1
Vimentin	VIM	Hs00958111_m1
CD44	CD44	Hs01075861_m1
NAD(P)H dehydrogenase (quinone) 1	NQO1	Hs02512143_s1
Heme Oxygenase 1	HMOX1	Hs01110250_m1
Albumin	ALB	Hs00609403_m1
CD68	CD68	Hs02836816_g1

**Table 3:** Method for dissociation.

Reagent	Enzyme Concentration or Quantity	Dissociation Time Day 3	Dissociation Time Day 9	Shaken by Hand
Liberase	1.3 U/mL	40 min	60 min	10× every 20 min
Accumax	900 µL	50 min	60 min	10× every 20 min
Accutase	900 µL	60 min	60 min	10× every 20 min

**Table 4:** Genes used for cell clustering.

Cell Type	Genes	Literature
HepaRG	*ALB* (>log 3 exp)*, KRT18, GSTA1, ABCC2, CYP3A5, CYP3A4, EGFR*	[3,54]
hTERT-HSC	*ACTA2, VIM, PDGFRB, PDGFRA, CD44*	[5,55,56]
THP-1	*CD33, CD64, CD56, LY75, LYST* (>1 exp)	[57,58]

## Data Availability

Single-cell RNA-seq data is available at http://doi.org/10.5281/zenodo.4708300.

## Supplementary Materials

Supplementary data are available online at https://www.mdpi.com/article/10.3390/ijms22094372/s1.
