# Supplementary material for: Single Cell Gene Expression Analysis in a 3D Microtissue Liver Model Reveals Cell Type-Specific Responses to Pro-Fibrotic TGF-β1 Stimulation
Source: Int J Mol Sci. 2021 Apr 22;22(9):4372. doi: 10.3390/ijms22094372 (PMC8122664; doi:10.3390/ijms22094372)
Supplement: Supplementary file 1 [file ijms-22-04372-s001.zip › Figure S1.pdf]

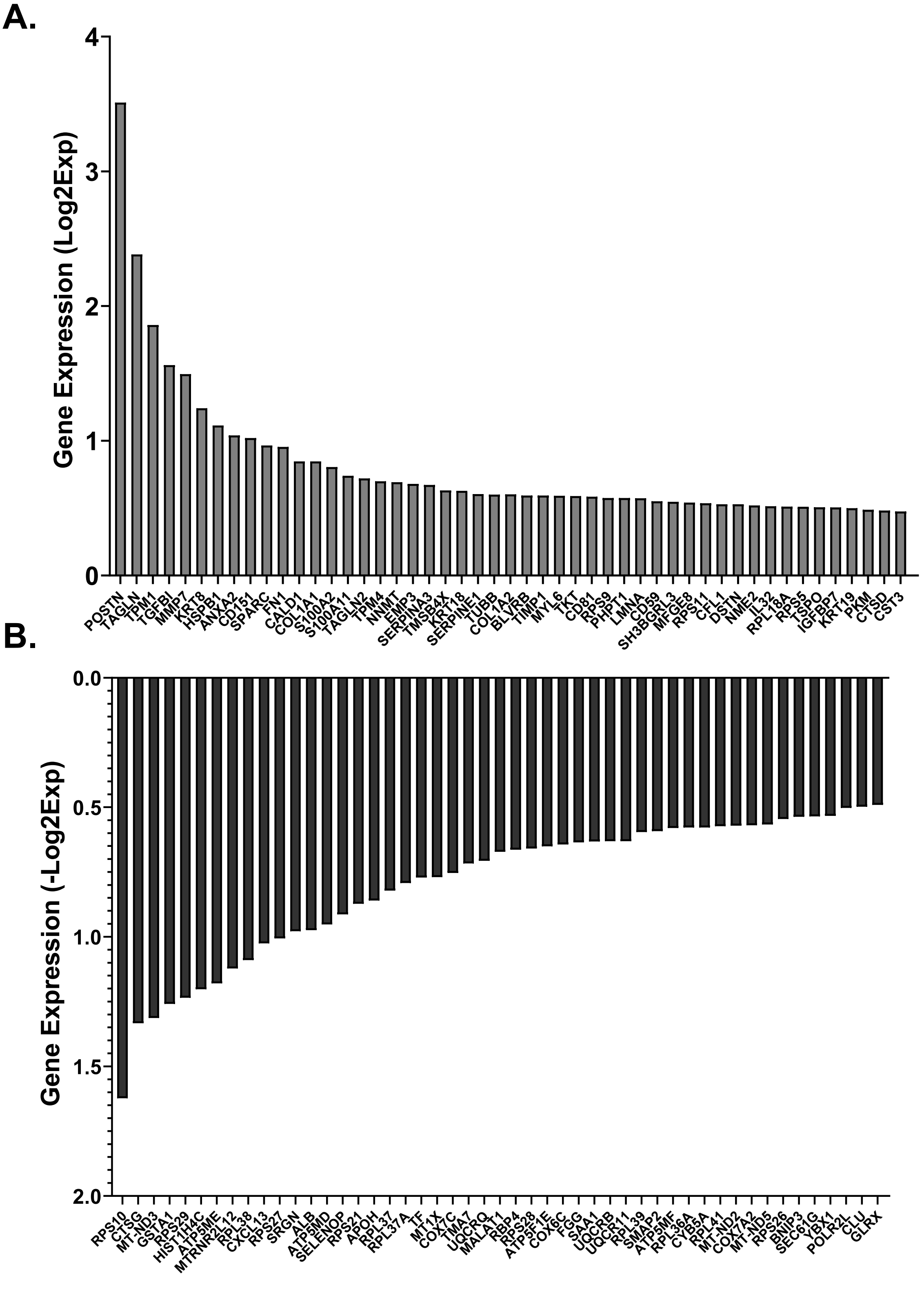

Figure S1. Top 50 differentially expressed genes in treated dissociated cell sample. Significantly upregulated (A) and downregulated (B) genes were identified for the unclustered TGF-β1 sample. Data are expressed as Log2Exp.
